# Macrophage Activity under Hyperglycemia: A Study of the Effect of Resveratrol and 3H-1,2-Dithiole-3-thione on Potential Polarization

**DOI:** 10.3390/molecules28165998

**Published:** 2023-08-10

**Authors:** Josué Manríquez-Núñez, Ofelia Mora, Francesc Villarroya, Rosalía Reynoso-Camacho, Iza Fernanda Pérez-Ramírez, Minerva Ramos-Gómez

**Affiliations:** 1 Departamento de Investigación y Posgrado de Alimentos, Facultad de Química, Universidad Autónoma de Querétaro, Centro Universitario S/N, Cerro de las Campanas, Querétaro 76010, Mexico; 2 Facultad de Estudios Superiores Cuautitlán, Universidad Nacional Autónoma de Mexico, Querétaro 76230, Mexico; 3 Department of Biochemistry and Molecular Biomedicine, Institute of Biomedicine of the University of Barcelona, 08007 Barcelona, Spain

**Keywords:** hyperglycemia, macrophage polarization, bioactive compounds, inflammation, resveratrol, 3H-1,2-dithiole-3-thione

## Abstract

Currently, research is focused on bioactive compounds with the potential to promote macrophage polarization with the aim of reducing the development of inflammatory-related diseases. However, the effect of bioactive compounds under oxidative-stress-induced hyperglycemia on macrophage polarization has been scarcely investigated. RAW 264.7 macrophages were incubated under standard (SG) or high glucose (HG) conditions and stimulated with lipopolysaccharide (LPS) (10, 60 and 100 ng/mL) to monitor macrophage polarization after resveratrol (RSV) or 3H-1,2-dithiole-3-thione (D3T) supplementation (2.5, 5, 10 and 20 µM). Under SG and HG conditions without LPS stimulation, RSV significantly decreased macrophage viability at the highest concentration (20 µM), whereas D3T had no or low effect. LPS stimulation at 60 and 100 ng/mL, under SG and HG conditions, increased significantly macrophage viability. Both RSV and D3T significantly decreased NO production in LPS-stimulated macrophages under HG condition, whereas only D3T increased GSH levels at 100 ng/mL and normalized MDA values at 60 ng/mL of LPS under HG condition. Under 60 ng/mL LPS stimulation and HG, mRNA IL-1 and IL-6 were higher. Interestingly, RSV decreased pro-inflammatory interleukins; meanwhile, D3T increased Arg1 and IL-10 relative expression. Overall, our results indicate that hyperglycemia plays a fundamental role in the modulation of macrophage-induced inflammation in response to bioactive compounds.

## 1. Introduction

Inflammation is generally considered as a pathological process promoted by conditions, such as infections, tissue injury, autoimmunity and in some cases exposure to several environmental factors [1]. Chronic metabolic diseases such as obesity and the frequently associated type 2 diabetes are concomitant with a systemic pro-inflammatory status in patients. Furthermore, excessive lipid accumulation in adipose tissue may promote pro-inflammatory mechanisms, favoring the migration and infiltration of elements of the immune system, such as macrophages [2]. This contributes to maintaining the inflammatory focus and enhancing the release of pro-inflammatory cytokines through NF-κB activation [3] and subsequently, if not resolved, the loss of function of the affected tissue in the long term [4,5]. In addition, macrophages represent a crucial cellular element of the innate immune system by playing roles such as critical regulators of tissue homeostasis [6].

Once macrophages mature and become tissue-resident cells, they could adopt either a pro-inflammatory phenotype (M1) or an anti-inflammatory phenotype (M2) [7]. Macrophage phenotypes are generally accepted as transient, reversible and modulated by a wide stimuli spectrum. M1 macrophages are the “classically activated” macrophages capable of dealing with pathogens and virus-infected cells or some pro-carcinogenic cells [8]. Also, M1 macrophages produce pro-inflammatory cytokines such as IL-1 and IL-6 and the inducible nitric oxide synthase (iNOS), which improves the effector function of M1 macrophages [9,10,11]. M2 macrophages represent an “alternatively” activated macrophage phenotype with anti-inflammatory activities that mostly promote inflammation resolution, tissue remodeling and wound healing expressing cytokines such as IL-10, as well as the enzyme Arg1 [12,13]. The ability of macrophages to alternate their phenotype between M1 and M2 is known as macrophage polarization. For example, Kang et al., evaluate the effect of Brassinin in a macrophage–adipocyte co-culture system, and the supplementation with this bioactive compound showed a significant reduction in the adipocyte lipid accumulation, as well as a decrease in the macrophage expression of pro-inflammatory interleukins such as IL-6, which suggest that this compound could inhibit obesity-induced inflammation [14]. This outcome suggests a polarization towards M2 which could be promoting the decrease in the accumulation of lipids within adipocytes.

In recent years, the main transcription factors involved in the polarization phenomenon have been proposed. NF-κB is the principal factor related to M1 macrophages, which promotes the transcription of pro-inflammatory proteins. On the other hand, Nrf2 has been related as a promising transcription factor due to its ability to neutralize oxidative stress, which is linked to a decrease in pro-inflammatory processes regulated by NF-κB, thereby allowing the activity of M2 macrophages [15]. Interestingly, both transcription factors are linked to the Keap1 repressor protein, which could function as a sensor pivot for the regulation of both proteins [16]. Due to the wide variety of molecular elements involved in the anti-inflammatory functions of macrophages, this polarization phenomenon remains unclear.

Currently, research is focused on the nutraceutical properties of different bioactive compounds, mainly from plant sources included in the diet, with the aim of reducing or preventing the development of pro-inflammatory-mediated non-transmissible chronic degenerative diseases. This is of great importance to elucidate the mechanisms involved, as well as the doses of consumption needed to exert the beneficial effects. The compounds supplemented in this study were chosen for their ability to elicit anti-oxidant/anti-inflammatory responses at the cellular level in RAW 264.7 macrophages by suppressing NF-κB activity mostly by mTOR (resveratrol) activation [12,17], as well as promoting the transcriptional activity of Nrf2 (3H-1,2-dithiole-3-thione) [18].

Resveratrol (RSV), a phenolic compound present in a wide variety of plants, is mainly produced under stress due to the attack of pathogens [19]. Several effects of RSV have been reported, such as antioxidant, anti-inflammatory, anti-carcinogenic, anti-aging, anti-diabetic and neuroprotective activities [20]. On the other hand, 3H-1,2-dithiole-3-thione (D3T) is the simplest member of the family of sulfur-containing diethylthiones; it can be found mainly in the *Brassicaceae* family and has been reported as a potent inducer of the antioxidant response defense due to its high capacity of Nrf2 activation [18].

The contribution of the modulation of macrophage activity under high glucose concentrations is poorly studied, as well as the effect of stimulation by different concentrations of bacterial lipopolysaccharide (LPS) under these glucose conditions. In this sense, the development of inflammatory-related diseases, such as overweight and obesity, is promoted due to a series of metabolic abnormalities, such as energy imbalance, increased oxidative stress, mitochondrial dysfunction, dysfunction of cellular elements of the tissue-resident immune system and chronic low-grade inflammation [21,22], in which the macrophage polarization could contribute to ameliorate the progression or promotion of those conditions. Therefore, the results obtained suggest that the effector functions of macrophages can be differentially modified by several factors, such as glucose concentration, LPS and the presence of various bioactive compounds; therefore, different phenotypes of macrophages can be achieved under different media conditions. In addition, our results provide insights into the potential impact of macrophage polarization over the progression of metabolic alterations like obesity and overweight through a better diet strategy, highlighting the need to explore further these mechanisms.

## 2. Results

### 2.1. Effect of RSV and D3T on Macrophage Viability under High Glucose (HG) Concentration and Stimulation with LPS

#### 2.1.1. Viability

**Figure 1:**
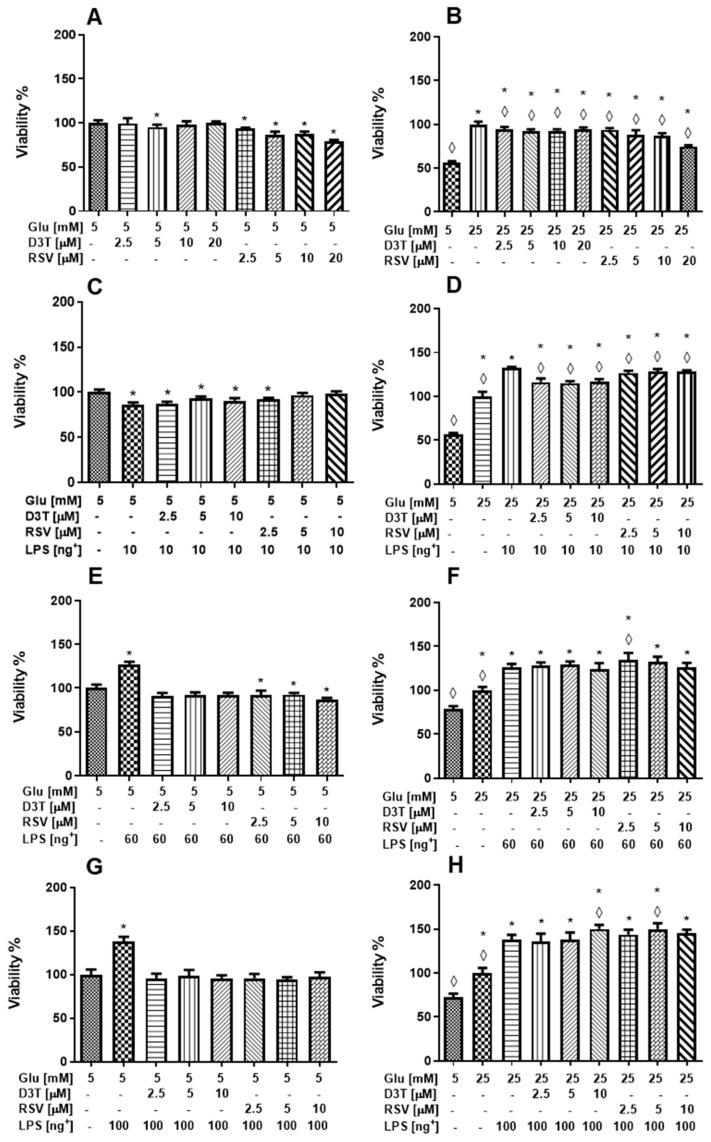
Effect of RSV and D3T supplementation on cell viability by MTT of RAW 264.7 cell line under glucose and LPS stimulation. Macrophage viability under (**A**) SG without LPS, (**B**) HG without LPS, (**C**) SG with 10 ng/mL of LPS, (**D**) HG with 10 ng/mL of LPS, (**E**) SG with 60 ng/mL of LPS, (**F**) HG with 60 ng/mL of LPS, (**G**) SG with 100 ng/mL of LPS and (**H**) HG with 100 ng/mL of LPS. * Indicates statistical difference (*p* < 0.01) with respect to control SG (5 mM) in (**A**,**C**,**E**,**G**), and ◊ indicates statistical difference with respect to HG without LPS in (**B**) and HG stimulated with LPS in (**D**,**F**,**H**). ^+^ LPS concentration (ng/mL).

Pro-inflammatory or M1 macrophages are commonly induced by microbial products, such as lipopolysaccharide (LPS) and cytokines, or by glucose [23]. Therefore, we aimed to evaluate macrophage viability by resveratrol and D3T under high glucose (HG) concentration and stimulation with LPS. Under standard glucose (SG) without LPS stimulation, both D3T and RSV decreased cell viability up to 4 and 21%, respectively (Figure 1A). On the other hand, macrophage viability significantly increased at HG (25 mM) without LPS stimulation (Figure 1B); however, D3T and RSV normalize cell viability by 8 and 25%, respectively. Furthermore, RSV at the highest concentration (20 μM) decreased macrophage viability by 20 and 25% at both SG and HG, respectively; therefore, both RSV and D3T at 20 µM were not further tested.

Figure 1C shows that macrophage viability in the SG group significantly decreased by 14% under LPS stimulation (10 ng/mL), as compared to that of SG without LPS stimulation. Interestingly, RSV exhibited a protective effect on macrophage viability at the highest concentrations (5 and 10 µM) under SG and LPS stimulation. On the other hand, LPS stimulation (10 ng/mL) under HG condition further increased macrophage viability by 32.5% (*p* < 0.01) (Figure 1D), whereas the addition of D3T and RSV also enhanced macrophage viability up to 16 and 28.7%, respectively (*p* < 0.01), as compared to 25 mM glucose-treated macrophages. Moreover, RSV exhibited a protective effect on macrophage viability at the highest concentrations under SG and HG conditions with LPS stimulation (Figure 1C,D), as compared to those without LPS stimulation (Figure 1A,B).

Figure 1E,G show that macrophage viability significantly increased at SG by 22.6 and 37.9 with 60 ng/mL and 100 ng/mL LPS stimulation, respectively. However, the addition of D3T and RSV at all concentrations normalized macrophage viability despite LPS stimulation. Similarly, macrophage viability at HG significantly increased by 47.6 and 65.7% with 60 and 100 ng/mL LPS, respectively (Figure 1F,H), which was further increased up to 53 and 77.4% by D3T and RSV supplementation, respectively, as compared to 25 mM glucose-treated macrophages.

Overall, under high glucose medium with LPS stimulation (10, 60 and 100 ng/mL), D3T and RSV increased macrophage viability as compared to 25 mM glucose-treated macrophages without LPS stimulation. On the contrary, D3T and RSV decreased or had no effect on macrophage viability at SG with and without LPS stimulation. These results were verified by the trypan blue technique with similar results to those obtained by the MTT assay (Figure S1).

#### 2.1.2. Cellular Protein Quantification

**Figure 2:**
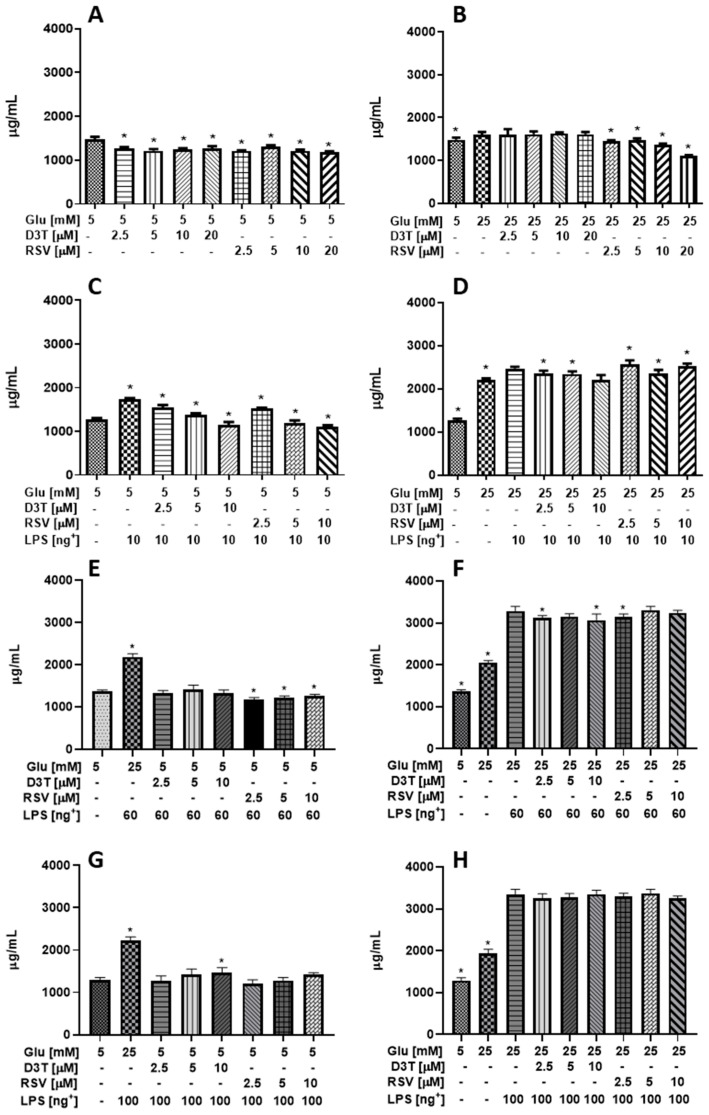
Effect of RSV and D3T supplementation on total cellular protein content by BCA of RAW 264.7 cell line under high glucose and LPS stimulation. The total protein content of macrophage by the BCA protein assay under (**A**) SG without LPS, (**B**) HG without LPS, (**C**) SG with 10 ng/mL of LPS, (**D**) HG with 10 ng/mL of LPS, (**E**) SG with 60 ng/mL of LPS, (**F**) HG with 60 ng/mL of LPS, (**G**) SG with 100 ng/mL of LPS and (**H**) HG with 100 ng/mL of LPS. * Indicates statistical difference (*p* < 0.01) with respect to control SG (5 mM) in (**A**,**C**,**E**,**G**), and indicates statistical difference respect to HG without LPS in (**B**) and HG stimulated with LPS in (**D**,**F**,**H**). ^+^ LPS concentration (ng/mL).

According to Figure 2A, D3T and RSV significantly decreased total cellular protein content under SG, with the greatest effect observed at the highest concentrations of RSV. In this regard, D3T and RSV significantly decreased total cellular protein content up to 18 and 19.5%, respectively (*p* < 0.01); although a “reset” effect on cellular protein content was observed for macrophages in high glucose (HG) medium (Figure 2B), the protein quantification also showed a progressive decrease with RSV treatments, reaching up to 15% for the highest concentration of RSV (20 μM) with respect to the control HG-treated macrophages (*p* < 0.01). Interestingly, we observed a protective effect with D3T at all concentrations tested; however, based on the effect of RSV at the highest concentration (20 μM), both RSV and D3T at 20 µM were not further tested under LPS stimulation.

By adding the stimulus of 10 ng/mL of LPS to the cell culture, the macrophage viability trend changed completely (Figure 1C). Under the SG condition, a 35.5% increase in cellular protein content over control without LPS was detected (*p* < 0.01); however, a progressive dose-dependent decrease in protein content was observed after D3T and RSV supplementation (Figure 2C). Furthermore, a significantly lower cellular protein content was observed at the highest concentration of D3T and RSV (10 µM) under LPS stimulation, as compared to that of SG-treated macrophages.

Interestingly, we observed similar results on total cellular protein contents after D3T and RSV supplementation under HG medium with LPS stimulation (10, 60 and 100 ng/mL; Figure 2D,F,H), as those obtained on macrophage viability (Figure 1D,F,H). Similar to macrophage viability outcome (Figure 1E,G), D3T and RSV decreased or had no effect on total cellular protein contents at SG medium with LPS stimulation (60 and 100 ng/mL, respectively).

### 2.2. Changes in NO Production by the Effect of RSV and D3T on RAW 264.7 Macrophages under Standard Glucose (SG) and High Glucose (HG) Conditions with LPS Stimulation

**Figure 3:**
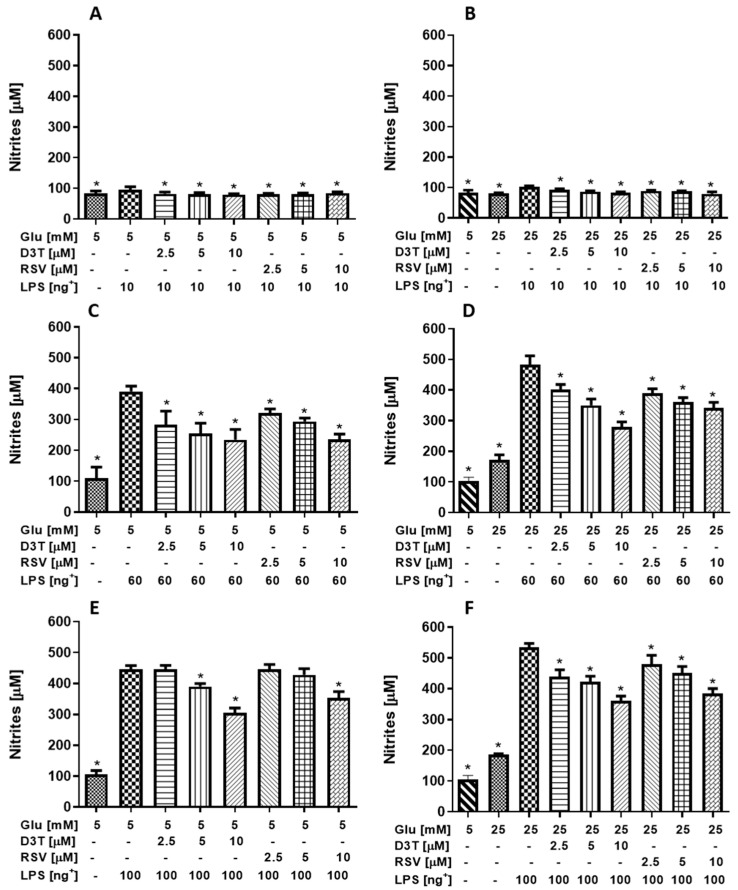
Effect of RSV and D3T supplementation on NO quantification of RAW 264.7 cell media under high glucose and stimulated with 10, 60 or 100 ng/mL of LPS. Quantification of nitrites at (**A**,**B**) SG and HG with 10 ng/mL of LPS, respectively; (**C**,**D**) SG and HG with 60 ng/mL of LPS, respectively; (**E**,**F**) SG and HG with 100 ng/mL of LPS, respectively. * Indicates statistical difference (*p* < 0.01) with respect to control LPS treatment without bioactive compound. ^+^ LPS concentration (ng/mL).

The detection of nitric oxide (NO) as nitrite content in a macrophage culture medium is a strategy to monitor the activation or attenuation of pro-inflammatory mechanisms by using a stimulus such as LPS. Therefore, the effectiveness of a treatment can be evaluated based on the inhibition or reduction in the concentration of nitrites derived from the experimental conditions (Figure 3).

Unexpectedly, LPS stimulation at 10 ng/mL increased NO production by 13 and 21% under SG and HG conditions, respectively, which was further decreased by up to 17% by D3T supplementation at SG condition (Figure 3A) and up to 22% by RSV at HG condition (Figure 3B), thus indicating a differential effect by D3T and RSV depending on the glucose concentration. On the other hand, LPS stimulation at 60 ng/mL increased NO production by 68 and 64% under SG (Figure 3C) and HG conditions (Figure 3D), respectively. As expected, a dose-dependent reduction in NO production was observed for both bioactive compounds under SG and HG conditions, with the greatest reduction of up to 36% with D3T under SG medium and up to 39% with RSV under HG condition. Similarly, LPS stimulation at 100 ng/mL increased NO production by 75 and 65% under SG (Figure 3E) and HG conditions (Figure 3F), respectively. Although a lower pronounced dose-dependent reduction in NO production was observed for both bioactive compounds under SG and HG conditions, as compared to those at 60 ng/mL, D3T supplementation showed the best dose–response effect with a maximum reduction of 33% at the highest concentration of 10 mM (*p* < 0.01, F = 8, 71, df = 71).

Overall, a better dose-dependent reduction in NO production by both bioactive compounds was observed at 60 and 100 ng/mL of LPS stimulation; therefore, LPS at 10 ng/mL was not further tested. In addition, as we observed an unfavorable effect on cell viability mainly with RSV at 20 µM under SG and HG conditions, this concentration for both bioactive compounds (RSV and D3T) was excluded in subsequent determinations; in the same way, as no significant differences were found in NO medium concentration at a 10 ng/mL of LPS concentration, it was decided not to use this treatment in subsequent evaluations.

### 2.3. Effect of the RSV and D3T on the Oxidative Stress Status (GSH and MDA) in RAW 264.7 under Standard (SG) and High Glucose (HG) Concentration and LPS Stimulation

**Figure 4:**
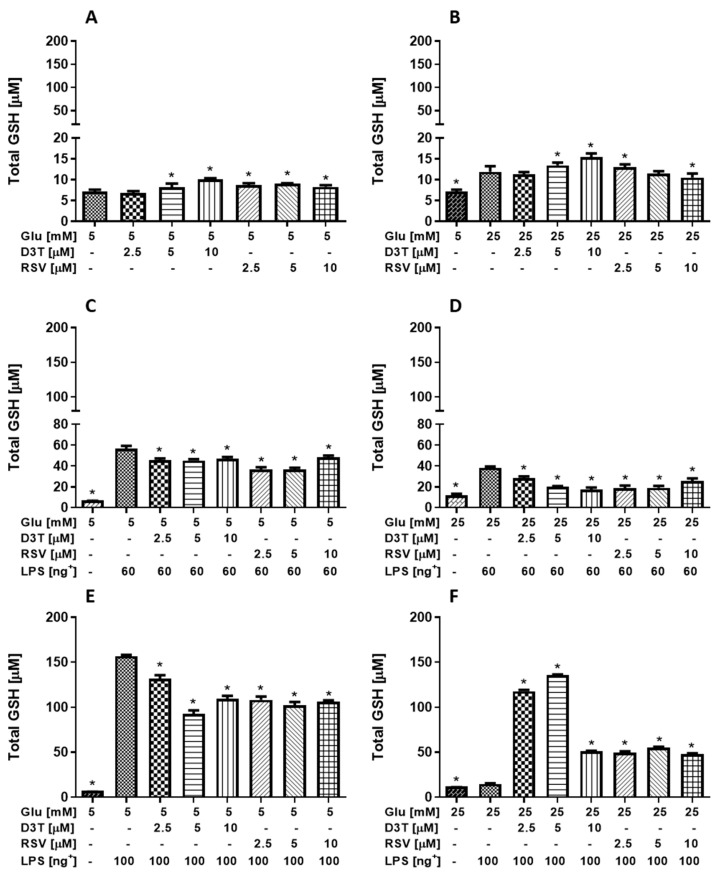
Effect of RSV and D3T supplementation on total intracellular GSH levels of RAW 264.7 cell line under high glucose and stimulated with 60 or 100 ng/mL of LPS. Total GSH content under (**A**) SG; (**B**) HG; (**C**) SG with 60 ng/mL of LPS; (**D**) HG with 60 ng/mL of LPS; (**E**) SG with 100 ng/mL of LPS and (**F**) HG with 100 ng/mL of LPS. * Indicates statistical difference (*p* < 0.01) with respect to control SG or HG condition with LPS stimulation and without bioactive compound. ^+^ LPS concentration (ng/mL).

As expected, D3T and RSV supplementation significantly increased the intracellular GSH content of macrophages under SG, with the highest increase of 39% with D3T in a dose-dependent manner (Figure 4A). Interestingly, the intracellular GSH content of macrophages under all treatments at HG was higher than those levels of macrophages under SG (Figure 4B). Similarly, D3T supplementation at the HG condition significantly increased GSH levels up to 30% in a dose-dependent manner; on the contrary, RSV supplementation significantly decreased intracellular GSH levels at the highest concentration (10 µM) with respect to the HG control.

The results obtained by the quantification of intracellular GSH of macrophages stimulated with 60 and 100 ng/mL of LPS at SG (Figure 4C,E) showed an increase of 7.3 and 21.9 times, respectively, compared with that of non-stimulated LPS control macrophages. Interestingly, intracellular GSH levels of macrophages supplemented with both D3T and RSV at all concentrations were significantly lower than those of their respective control 60 and 100 ng/mL of LPS-stimulated macrophages under SG (Figure 4C,E); however, GSH levels were higher than those of macrophages without LPS stimulation (Figure 4A).

At high glucose conditions, the intracellular GSH level of macrophages stimulated with 60 ng/mL of LPS (Figure 4D) increased by 3.2 times, compared with that of non-stimulated LPS control macrophages (Figure 4B). Similarly, GSH levels of macrophages supplemented with both D3T and RSV at all concentrations were significantly lower than that of 60 ng/mL of LPS-stimulated macrophages under HG, but still remained higher than those of GSH levels of non-stimulated LPS control macrophages.

Unexpectedly, the GSH level of macrophages stimulated with 100 ng/mL of LPS under HG condition (Figure 4F) dropped closer to the value of non-stimulated LPS control macrophages. Furthermore, both D3T- and RSV-treated macrophages showed higher GSH levels than that of control LPS-stimulated macrophages under HG, with the highest levels at 2.5 and 5 µM of D3T.

**Figure 5:**
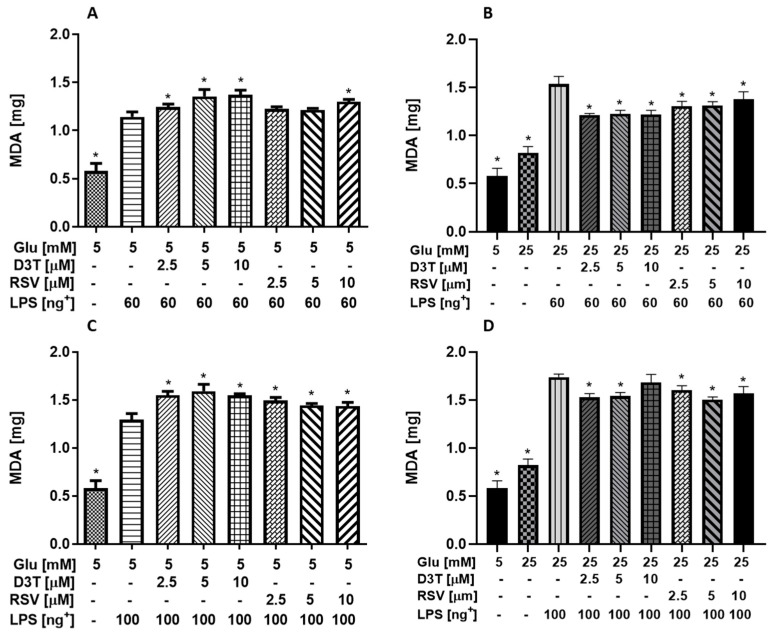
Effect of RSV and D3T supplementation on MDA levels of RAW 264.7 cell line medium under SG and HG stimulated with LPS. MDA content at (**A**) SG with 60 ng/mL of LPS; (**B**) HG with 60 ng/mL of LPS; (**C**) SG with 100 ng/mL of LPS and (**D**) HG with 100 ng/mL of LPS. * Indicates statistical difference (*p* < 0.01) with respect to control SG with LPS for A and C; HG with LPS for B and D. ^+^ LPS concentration (ng/mL).

Thiobarbituric acid reactive substance assay (TBARS) is performed to measure lipid peroxidation in a variety of cell lines, tissue samples and biological fluids. The assay is based on the detection of malondialdehyde (MDA), the major lipid oxidation product that is often considered a reference of the oxidative stress level in the sample. In this regard, RAW 264.7 macrophages stimulated with 60 and 100 ng/mL of LPS or under HG showed higher MDA levels (96, 121 and 40.7%, respectively) than that of non-LPS-stimulated macrophages under SG condition (Figure 5A), which was further increased with 60 and 100 ng/mL of LPS under HG (164 and 198.8%, respectively).

Unexpectedly, significant increases in MDA levels up to 14 and 9% were observed with 60 ng/mL of LPS and up to 17.4 and 156.7% with 100 ng/mL of LPS with D3T and RSV, respectively, under SG conditions (Figure 5A,C). On the contrary, MDA levels were significantly lower with D3T and RSV at 60 and 100 ng/mL of LPS under HG conditions (Figure 5A,C), but still remained higher than those MDA levels of stimulated LPS control macrophages.

Overall, the results suggest that MDA levels increased after D3T or RSV supplementation under SG conditions at 60 and 100 ng/mL of LPS. However, MDA values were lower after D3T or RSV supplementation under HG conditions at 60 and 100 ng/mL of LPS.

### 2.4. RSV- and D3T-Mediated Suppression of LPS-Induced Pro-Inflammatory Responses in RAW 264.7 Cells under Hyperglycemia

According to the results obtained in NO, GSH and MDA quantification under LPS stimulation with 100 ng/mL, no significant decreases were observed at the lowest concentrations of bioactive compounds (2.5 µM), neither in the quantification of MDA under HG conditions nor at the same concentration of LPS; therefore, it was decided not to evaluate the 100 ng/mL LPS treatments for the subsequent determinations of gene relative expression.

**Figure 6:**
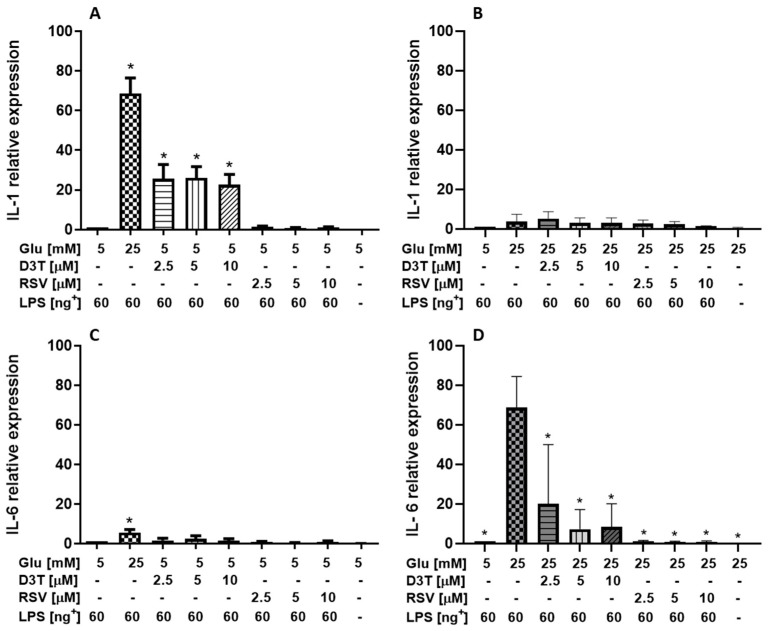
Relative gene expression of pro-inflammatory genes by the effect of RSV and D3T supplementation on RAW 264.7 cell line under glucose and LPS stimulation (60 ng/mL): (**A**,**B**) relative expression of IL-1 in SG and HG with 60 ng/mL of LPS, respectively; (**C**,**D**) relative expression of IL-6 in SG and HG with 60 ng/mL of LPS, respectively. * Indicates statistical difference (*p* < 0.01) with respect to control: (**A**,**C**) SG condition plus LPS without bioactive compound; (**B**,**D**) HG condition plus LPS without bioactive compound. ^+^ LPS concentration (ng/mL).

The results of the relative gene expression (mRNA levels) of pro-inflammatory cytokines are shown in Figure 6. In general, D3T decreased the relative expression of IL-1 by up to 64% with respect to the LPS control, while RSV decreased the expression to levels similar to those of the SG control without LPS stimulation (Figure 6A). This trend is not observed for macrophages under HG condition, where the supplementation with the bioactive compounds in general does not show a significant decrease with respect to LPS-stimulated macrophage control (Figure 6B). On the contrary, a significant induction of IL-1 (67 times-fold change, *p* < 0.01) in HG-treated macrophages stimulated with 60 ng/mL of LPS was observed with respect to the control SG-treated macrophages (Figure 6A). Unexpectedly, D3T supplementation significantly increased the relative expression of IL-1 by up to 67%. However, RSV slightly induced the relative expression of IL-1 to values similar to those of SG-control macrophages stimulated with 60 ng/mL of LPS (*p* > 0.05). On the contrary, under HG condition (Figure 6B), we observed a reduction of up to 18 and 64% in the relative expression of IL-1 by D3T and RSV, respectively, except for the significant folding of 6.1 times after 2.5 µM of D3T.

On the other hand, the relative expression of the pro-inflammatory IL-6 of HG-treated macrophages stimulated with 60 ng/mL of LPS was only 5.5 times more than that of SG-treated macrophages (*p* < 0.01, Figure 6C). Similarly, D3T supplementation slightly increased the relative expression of IL-6 up to 0.6 times under SG (Figure 6C) and significantly decreased it by 6.3 times under HG (Figure 6D). However, RSV decreased the relative expression of IL-6 to values similar (up to 0.3 times) to those of SG-control macrophages and significantly decreased it by up to 23% under HG-control macrophages stimulated with 60 ng/mL of LPS (*p* < 0.01) (Figure 6C,D). These results are of major relevance since the relative expression of the pro-inflammatory IL-6 of HG-treated macrophages stimulated with 60 ng/mL of LPS was 68 times more than that of unstimulated HG-treated macrophages (*p* < 0.01, Figure 6C).

Overall, the relative expression of the pro-inflammatory cytokines IL-1 and IL-6 were similar to control values under SG and significantly lower under HG conditions after RSV supplementation in LPS-stimulated macrophages.

### 2.5. RSV- and D3T-Mediated Induction of Anti-Inflammatory Responses and M2 Molecular Markers of LPS-Stimulated RAW 264.7 Cells under Hyperglycemia

**Figure 7:**
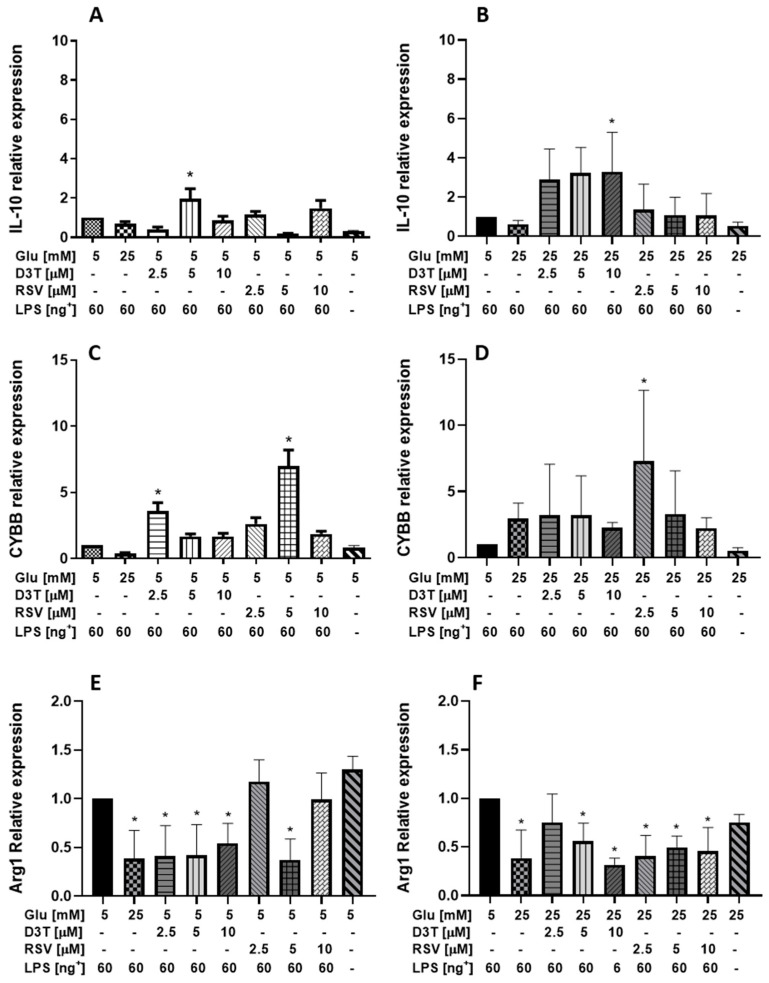
Relative gene expression of anti-inflammatory cytokine IL-10, M1 biomarker CYBB and M2 biomarker Arg1 after RSV and D3T supplementation of RAW 264.7 cell line under glucose and LPS stimulation (60 ng/Ml). (**A**,**B**) Relative expression of IL-10 under SG and HG with 60 ng/mL of LPS, respectively; (**C**,**D**) relative expression of CYBB under SG and HG with 60 ng/mL of LPS, respectively; (**E**,**F**) relative expression of Arg1 under SG and HG with 60 ng/mL of LPS, respectively. * Indicates statistical difference (*p* < 0.01) with respect to SG-control condition plus LPS stimulation without bioactive compound for (**A**,**C**,**E**) and for (**B**,**D**,**F**) HG-control condition plus LPS stimulation without bioactive compound. ^+^ LPS concentration (ng/mL).

Regarding IL-10 (Figure 7), the expression of this interleukin without LPS stimulation was 70 and 50% lower compared with those under SG and HG LPS-stimulated control macrophages, respectively; meanwhile, there are no significant differences in the relative expression between the SG- and HG-treated macrophages stimulated with 60 ng/mL of LPS without bioactive compounds. Interestingly, D3T 5 µM supplementation was the only treatment that significantly increased up to 6 times the relative expression of IL-10 compared to that of SG-control macrophages (*p* < 0.01); however, we did not observe a dose–response effect for both compounds under SG (Figure 7A). On the contrary, a dose–response induction in the relative expression of L-10 was observed after D3T supplementation under HG conditions, being statistically significant at the highest concentration (*p* < 0.01). Although not statistically different, the relative expression of IL-10 after RSV supplementation was higher than that of HG-control macrophages stimulated with 60 ng/mL of LPS (Figure 7B).

On the other hand, we did not observe any dose–response in the relative expression of the M1 type marker CYBB (Figure 7C,D), which has been proposed as a novel marker due to its participation in the generation of ROS and in the pro-inflammatory functions of M1 macrophages [24], for both compounds under SG and LPS (60 ng/mL) stimulation. Unexpectedly, the relative expression of CYBB was significantly higher up to 3.5 and 7 times after D3T 2.5 µM and RSV 5 µM supplementation, respectively (Figure 7C), under SG and LPS (60 ng/mL) stimulation. Moreover, the relative expression of CYBB was significantly higher up to 6 times after RSV 2.5 µM supplementation (Figure 7D) under HG condition with 60 ng/mL of LPS. Although Arg1 has been identified as a marker of the M2 phenotype [25,26], under the evaluated conditions, in general, we found significant decreases in the relative expression after D3T supplementation in the presence of LPS of up to 58% similar to those of the HG control which are up to 64% (Figure 7E); in contrast, under SG and LPS stimulation, only a slight increase (up to 11%) in the expression of Arg1 could be determined after 2.5 µM of RSV. On the other hand, it is possible to notice the general trend of decrease in the Arg1 relative expression with D3T and RSV treatments (65 and 58%, respectively) in macrophages under the HG medium. However, D3T at 2.5 µM was the only treatment that shows a less decrease in the relative expression of Arg1 (around 26%) similar to that observed in macrophages in HG without LPS (Figure 7F).

Overall, the relative expression of IL-10 and the M1-type marker CYBB was higher under HG conditions with 60 ng/mL of LPS. Unexpectedly, both mRNA levels were higher after LPS stimulation and HG medium, as compared to the relative expression under SG and HG without LPS stimulation.

### 2.6. Changes in Macrophage Morphology Mediated by LPS Stimulation under SG and HG in the Presence of RSV or D3T

**Figure 8:**
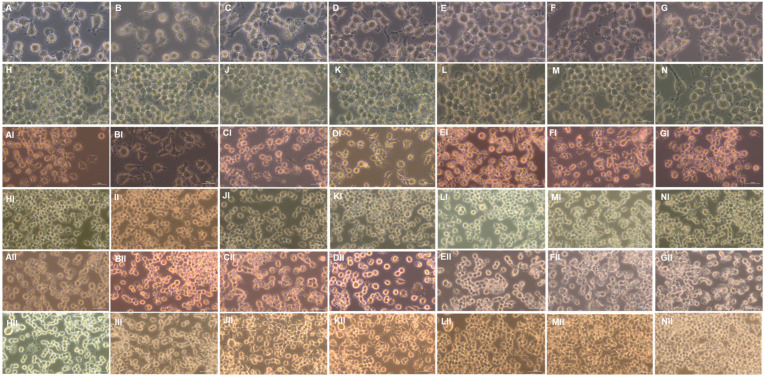
Effect of the bioactive compounds D3T and RSV on the morphology and confluence of the RAW 264.7 cell line. SG condition with 10 ng/mL of LPS stimulation: (**A**,**B**) 2.5 µM of D3T; (**C**) 5 µM of D3T; (**D**) 10 µM of D3T; (**E**) 2.5 µM of RSV; (**F**) 5 µM of RSV; (**G**) 10 µM of RSV. HG condition with 10 ng/mL of LPS stimulation: (**H**) control; (**I**) 2.5 µM of D3T; (**J**) 5 µM of D3T; (**K**) 10 µM of D3T; (**L**) 2.5 µM of RSV; (**M**) 5 µM of RSV; (**N**) 10 µM of RSV. The order of the treatments from (**AI**–**NI**) corresponds to treatments with a stimulus of 60 ng/mL of LPS, respectively. The order of the treatments from (**AII**–**NII**) corresponds to treatments with a stimulus of 100 ng/mL of LPS, respectively.

In general, the images obtained with the inverted microscope (Figure 8) showed that RAW 264.7 macrophages incubated under SG medium and stimulation with LPS generate more extensive changes in the membrane (Figure 8A–G,AI–GI,AII–GII) than those in HG medium (Figure 8H–N,HI–NI,HII–NII), which exhibited a much more rounded membrane. In the same way, it is possible to observe that RSV and D3T supplementation at all concentrations modified the macrophage membrane under SG conditions; however, the same effect is not seen in macrophages treated under HG conditions. In addition, LPS stimulation at higher concentrations increased the extension of the membrane under SG, being the macrophages treated with D3T under HG and 100 ng/mL of LPS with similar characteristics to those incubated under SG without LPS stimulation. This could indicate that this bioactive compound could be stimulating macrophage membrane activation under HG conditions by inducing Nrf2; this improvement in membrane extensions could be an important characteristic because it would be an indicator of a higher phagocytic capacity under HG conditions, which would contribute significantly to the role of macrophages as an element of the innate immune system in different pathologies.

## 3. Discussion

It is known that macrophages under a HG stimulus even for short periods tend to polarize into M1-type macrophages due to overstimulation of glycolysis, in addition to the induction of the pro-inflammatory response via NF-κB [27], probably mediated by oxidative stress. On the other hand, M1 and M2 diversity subsets are not firmly differentiated, implying that macrophages undergo a dynamic transition between differential states, which is determined by the microenvironment, thus impacting the dual-functional properties of M1/M2 macrophages. In this sense, we aimed to evaluate the change in the characteristics of macrophages cultured in SG and HG media with or without increasing LPS stimulation in order to simulate the pathological condition of obesity. In this study, D3T and RSV were supplemented due to their impact and ability to reduce inflammation through important pathways involved in the regulation of cellular homeostasis, such as Nrf2 and Akt/mTOR, respectively [17,28], and to promote M2 macrophage polarization. Therefore, the high glucose and pro-inflammatory factors, in addition to the activation of known pathways by the compounds applied, RSV (mTOR) and D3T (Nrf2), provided us information on how these potential M2 macrophage subtypes could participate in the development and progression of inflammatory-related pathologies.

Initially, we found a significant decrease in RAW 264.7 macrophage cell viability at the highest concentration of RSV (20 µM) under SG and HG conditions. This differs from that previously reported for macrophages supplemented with RSV at 30 µM under SG conditions [12,29,30]. Although not reported, primary peritoneal macrophages isolated from mice and RAW 264.7 cells were treated with up to 100 µM of D3T without any negative effect on macrophage viability [31,32]. Similarly, we could not observe any adverse effect of D3T on macrophage viability under SG and HG conditions.

Previous evaluations related to the effects of RSV and D3T on macrophage cell viability under HG and LPS stimulation are scarce. In this study, we report in general increases in the viability of macrophages under HG conditions, which is supported by studies carried out in other cell lines such as VSMCs and VEGF due to an alteration in VEGF transcription linked to an increase in oxidative stress [33]. Moreover, this could also modify the macrophages stimulated with LPS by the induction of antioxidant systems by the bioactive compounds evaluated increasing their viability. Recently, a significant viability reduction (around 12%) has been reported with RSV (20 µM) under HG conditions and 10 ng/mL of LPS stimulation [34]. Similarly, RAW 264.7 cells were pretreated with 0–100 µM of D3T and then exposed to LPS (10 ng/mL) where macrophage cell viability was not reported [31], suggesting a low cytotoxic potential of D3T unlike RSV; however, the bioactivity under increasing LPS stimulation has not been determined. To our knowledge, this is the first report about the effect of D3T on cell viability under HG and LPS-stimulated macrophages. In addition, there was a positive correlation between the MTT viability assay and total cellular protein content (*p* < 0.01); therefore, the protein content could also be used as a reference to determine viability intervals. Similar to our study, RAW 264.7 macrophage proliferation as well as total protein mass were determined under 100 ng/mL LPS stimulation, where an increase in the protein concentration after 24 h of incubation with LPS was reported [35].

Saleh et al. [34] did not find a significant reduction in NO production under similar conditions and supplementation with RSV (5–20 µM), which differs from the results obtained in this study. Recently, Zhu et al. [31] reported a NO dose-dependent reduction of up to 20% mediated by D3T (0–25 µM) under LPS stimulation (10 ng/mL). Similar to the previous studies with RSV, this reduction in NO concentration was achieved by 24 h-pretreatment with D3T before induction with LPS. Although no results on NO quantification in the culture medium are shown by Yu et al. [12], this could be related to low NO levels due to the inhibition in the iNOS stimulation after 30 µM of RSV and LPS (1 µg/mL)-stimulated RAW 264.7 macrophages. In addition, intracellular levels of GSH in RAW 264.7 macrophages after supplementation with compounds, such as N-acetyl-l-cysteine, bromosulfophthalein and D3T, under SG without LPS stimulation [31,36,37] are consistent with the results obtained in our study after D3T or RSV supplementation without LPS stimulation. As expected, D3T induces in general a higher GSH concentration in most of the conditions evaluated. The bioactivity of RSV under HG conditions showed a significant decrease in NO concentration, mainly due to the scavenging of ROS by RSV [38]. Although these effects have been evaluated in several in vivo and in vitro models, the influence of increasing concentrations of LPS and the participation of NF-κB in macrophage activity are not entirely clear. The importance of a broad screening of M1/M2 macrophage characteristics is essential to determine the feasibility of bioactive compounds in the treatment or prevention of inflammatory-mediated diseases.

In this study, RSV supplementation lowered MDA concentration under SG conditions, whereas D3T had the best results under HG conditions, which would suggest that the Nrf2 induction by D3T could contribute to lower tissue inflammation under hyperglycemia. The supplementation with RSV derivatives, such as 3,3′,4,5′-tetramethoxy-trans-stilbene and 3,4′,5-trimethoxy-trans-stilbene, significantly decreased intracellular MDA levels up to 50%, regardless of the concentration of both derivatives (10 and 50 µM), in LPS (1 µg/mL)-stimulated RAW 264.7 macrophages under HG conditions [39]. In this sense, Zhu et al. [31] suggested that bioactive compounds such as 3H-1,2-Dithiole-3-thione can reduce the pro-inflammatory signaling via NF-κB in macrophages cultured under standard conditions and low inflammation (10 ng/mL of LPS) by mitigating the oxidative stress; however, these effects have not been reported under hyperglycemia conditions. The increase in MDA concentration (Figure 5A,C) under SG conditions could be related to the augmentation in GSH concentration under the same conditions (Figure 4C,E), because it has been observed that the formation of MDA from arachidonic acid by the COX2 enzyme depends on high levels of GSH [40]. Moreover, it is suggested that this relationship depends on the GSH concentration but not necessarily in a concentration-response way, which could also explain the behavior of the MDA concentration in HG.

The levels of the relative expression of IL-1 and IL-6 mRNA in SG-LPS (60 ng/mL)-stimulated macrophages with RSV were higher than those reported with a LPS stimulus (10 ng/mL) under SG [34,41], but similar to that of macrophages incubated with RSV analogues (pterostilbene), showing a significant decrease by up to 50% in the relative expression of IL-1 mRNA compared to control macrophages [42]. In our study, D3T supplementation decreased the relative expression of IL-1 by up to 71% compared to that under SG-LPS (60 ng/mL) condition. D3T treatment also led to a concentration-dependent suppression of LPS-induced interleukin-1beta (IL-1β) and NO release in RAW 264.7 cells at SG [39].

Moreover, the relative expression of IL-10 mRNA under HG conditions agrees with that reported by Chung et al. [43], in differentiated macrophages under HG with LPS stimulation (100 ng/mL). In general, treatments with D3T under hyperglycemia showed a greater induction in the relative expression of IL-10 compared to that treated with RSV, suggesting that regulation under Nrf2 stimulation could be more efficient than mTOR induction. Similarly, Palacz-Wrobel et al. [30] reported a decrease of up to 36% in IL-10 expression after RSV supplementation (30 µM) under SG and LPS stimulation (100 ng/mL). On the other hand, Yu et al. [12] observed a poor IL-10 mRNA induction using similar RSV concentration under SG-LPS (1 µg/mL) condition. These results are consistent with those shown in our study (Figure 7A), where non-statistical difference in the relative expression of IL-10 at different concentrations of RSV was observed with respect to control macrophages. To our knowledge, there is no information available about D3T effects on the relative expression of IL-10; this is of major relevance since D3T at a 5 µM concentration significantly increased IL-10 levels under similar conditions described before.

Besides that, Arg1 is an important gene in M2 activity, we did not find a significant induction of this gene under the experimental conditions evaluated. On the contrary, we observed significant reductions in the relative expression of Arg1 of up to 58 and 62% under SG conditions after D3T and RSV supplementation, respectively, attaining similar levels with 2.5 µM of RSV, to those of control macrophages without LPS. Likewise, only D3T at 2.5 µM supplementation under HG conditions significantly decreases the expression of Arg1 similar to that of the control without LPS; moreover, a significant decrease in the Arg1 expression for the rest of the treatments was observed in the order of 66 to 59% for D3T and RSV, respectively. This behavior of Arg1 agrees with that reported by Figueiredo et al. [44], where it suggests that the induction of this gene by bioactive compounds could be affected by the microenvironment to which macrophages are subjected.

Finally, the morphology changes of RAW 264.7 macrophages under HG, LPS stimulation and bioactive compounds supplementation were noticeable throughout the study. In this regard, images obtained on the cell morphology by microscopy showed several similarities with those reported by Venter et al. [35], in which a pivotal function of high glucose concentration in the presence of LPS suggested a possibly over-activation of glycolysis, thus altering the morphodynamics of macrophages.

Most of the previous studies about the effect of RSV cited in this study have been performed under SG and low (10 ng/mL) or very high levels (1 µg/mL) of LPS. According to our results, most of the parameters behaved differently for each bioactive compound once the macrophages were exposed to an HG condition and increasing levels of LPS stimulation. In the case of RSV, most studies point to an inhibition of NF-κB activation through AKT/mTOR, which would explain the decrease in the relative expression of pro-inflammatory genes (IL-1 and IL-6), but not for the anti-inflammatory elements under SG conditions; meanwhile, most of the studies confirm that the anti-inflammatory effects of D3T are reached through Nrf2 induction under SG conditions, but these outcomes have not been further explored under HG conditions. Moreover, an important factor in the development of inflammatory-related disease is the increase in oxidative stress, which generates an increase in pro-inflammatory signals [15]. In this sense, compounds such as D3T through Nrf2 stimulation could mitigate oxidative stress and therefore the pro-inflammatory response via NF-κB; this mechanism could be partially activated by RSV in conjunction with other pathways such as Akt/mTOR for the neutralization of oxidative stress. In this regard, although D3T does not reduce the relative expression of the pro-inflammatory interleukins IL-1 and IL-6 in the same ranges as those reached by RSV, it significantly promotes IL-10 expression contributing to tissue remodeling and the decrease of systemic inflammation under hyperglycemia conditions. This outcome could be considered an important therapeutic focus in the treatment of conditions, such as obesity and overweight, where tissue atrophy derived from chronic inflammation occurs as the disease evolves.

## 4. Materials and Methods

### 4.1. Cell Culture

RAW 264.7 cells were obtained from American Type Culture Collection (ATCC, Rockville, MD, USA). RAW 264.7 cells were cultured in Dulbecco’s Modified Eagle Medium (DMEM) at standard glucose concentration (5 mM, SG) and high glucose concentration (25 mM, HG). Cells were supplemented with 10% fetal bovine serum (FBS), 100 U/mL penicillin and 100 μg/mL streptomycin and incubated at 37 °C under 5% CO_2_.

### 4.2. MTT and Trypan Blue Assay

RAW 264.7 cells were cultured in 96-well cell culture plates at a density of 2 × 10^4^ cells/mL under SG or HG and supplemented with different concentrations (2.5, 5, 10 and 20 μM) of D3T or RSV (Sigma-Aldrich, St. Louis, MI, USA) and LPS stimulation (Sigma-Aldrich, St. Louis, MI, USA) at 10, 60 or 100 ng/mL for 24 h. These concentrations were chosen based on preliminary works. Therefore, in order to carry out all experiments under appropriate/optimal cell viability conditions, both MTT and Trypan blue assays were determined. According to this, MTT is based on the reduction of a yellow tetrazolium salt (3-(4,5-dimethylthiazol-2-yl)-2,5-diphenyltetrazolium bromide or MTT) to purple formazan crystals by the mitochondrial activity of metabolically active cells [45]. After 24 h incubation, 100 μL of MTT solution 1 mg/mL (Sigma-Aldrich) was added to the medium according to the manufacturer’s protocol, and the cells were incubated for 2 h. The MTT-containing supernatant was removed and the resulting formazan crystals were dissolved in DMSO. The MTT signal was determined by measuring the absorbance at 570 nm with a Varioskan microplate reader (Thermo Scientific^TM^, Waltham, MA, USA). Viability is expressed as a percentage of each treatment relative to the control macrophages according to the manufacturer’s instructions. Similarly, Trypan blue stain exclusion test is based on the concept that viable cells do not incorporate impermeable dyes (such as Trypan blue); however, dead cells are permeable and absorb the dye [46]. Therefore, after 24 h of incubation with treatments, cells were carefully washed with warm PBS and proceeded to detach; afterwards, the 0.4% Trypan blue solution (Sigma-Aldrich) was added and allowed to incubate for 5 min and proceeded to cell counting in a Neubauer chamber. Cellular viability is expressed as a percentage of each treatment relative to the control macrophages according to the manufacturer’s instructions.

### 4.3. Total Cellular Protein Determination

RAW 264.7 cells were cultured in 96-well cell culture plates at a density of 2 × 10^4^ cells/mL under SG or HG supplemented with different concentrations (2.5, 5, 10 and 20 μM) of D3T or RSV and LPS stimulation at 10, 60 or 100 ng/mL for 24 h. After the incubation period, the quantification of total protein was measured for the attached cells (living cells) by using the Pierce™ BCA Protein Assay Kit (Thermo Scientific^TM^) according to the manufacturer’s instructions. Total cellular protein concentration was determined by extrapolation with an albumin calibration curve (0 to 2000 mg/mL).

### 4.4. Nitric Oxide Production

Nitric oxide (NO) formation was detected based on the accumulation of nitrites, an indicator of NO synthesis, in the culture medium. RAW 264.7 cells were seeded at 2 × 10^4^ cells/mL in a 96-well plate under SG or HG and supplemented with different concentrations (2.5, 5, 10 and 20 μM) of D3T or RSV and LPS stimulation at 10, 60 or 100 ng/mL for 24 h. After the incubation period, NO concentration was determined by measuring the amount of nitrite produced in the cell culture supernatant using the Griess reagent. The absorbance measurement at 540 nm was performed using a Varioskan microplate reader (Thermo Scientific), and then the results were extrapolated using a calibration curve of sodium nitrite (0 to 1000 µM).

### 4.5. TBARS Assay for MDA Detection

The TBARS is widely used to determine lipid peroxidation in biological samples as an indicator of oxidative stress [47]. For this, the reagent was prepared prior to the analysis of the samples, which consisted of 15% trichloroacetic acid, 0.375% thiobarbituric acid and 2.1% hydrochloric acid (V/V/V) in distilled water. The reaction mixture was prepared with one part of the sample plus 5 parts of TBARS reagent; then, samples were placed in an ultrasound bath with distilled water at 90 °C for 20 min. Once the incubation period was finished, the samples were centrifuged at 10,000 rpm for 10 min. Then 270 µL of supernatant was recovered and placed in a 96-well plate and the absorbance was measured at 532 nm in a spectrophotometer. The results are expressed by extrapolation with a calibration curve of MDA (mg).

### 4.6. Total GSH Levels

For the evaluation of total intra-cellular thiol levels, cell pellets were re-suspended in 1 mL of TCA (20%), centrifuged at 10,000 rpm for 10 min and the aliquots of the supernatants were adjusted to pH 7.5–8.0 with K_2_CO_3_. Total cellular GSH was assayed by the modified Griffith method in which reduced GSH was sequentially oxidized by 5,5′-dithiobis-(2-nitrobenzoic acid) (DTNB) to GSSG. The rate of DTNB formation is monitored at 412 nm and the glutathione level was calculated using a standard curve (0 to 200 µM).

### 4.7. RNA Isolation and qPCR Analysis

The quantification of the relative expression of mRNA, as a strategy to determine the potential polarization under the experimental conditions evaluated, was performed as follows. Total cellular RNA was isolated from RAW264.7 after the incubation with the bioactive compounds and LPS treatments with TRIzol reagent (TRIzol^TM^, Invitrogen^TM^, Waltham, MA, USA) and then reverse transcribed into cDNA with M-MLV^®^ RT kit from Promega (Promega, Madison, WI, USA). Afterwards, real-time PCR was performed with FastStart SYBR^®^ Green Master (Roche, Basel, Switzerland). Analysis was performed using the StepOne RealTime PCR System (Applied Biosystems, Waltham, MA, USA) with the following program: pre-heat at 95 °C for 10 min; denaturation at 95 °C for 15 s; annealing at 60 °C for 1 min; melting curve 95 °C for 15 s; according to the manufacturer’s instructions. The housekeeping 18S rRNA and RPL0 genes were used for the normalization method as part of the ΔΔCt relative expression analysis. The sequences of the primers used for the analysis are given in Supplementary Table S1.

### 4.8. Microscopy

The microscopy images were obtained with an inverted Axio Observer II Zeiss microscope (Zeiss, Oberkochen, Germany), using the PHII filter and the 20× objective.

### 4.9. Statistical Analysis

Results are expressed as the means ± standard deviation (SD) of three independent experiments (*n* = 3). Statistical analyses for the different determinations were performed by applying one-way ANOVA followed by Dunnett’s test for comparisons of means (*p* < 0.01) using Prism GraphPad version 8 software (GraphPad Software 2022, San Diego, CA, USA).

## 5. Conclusions

**Figure 9:**
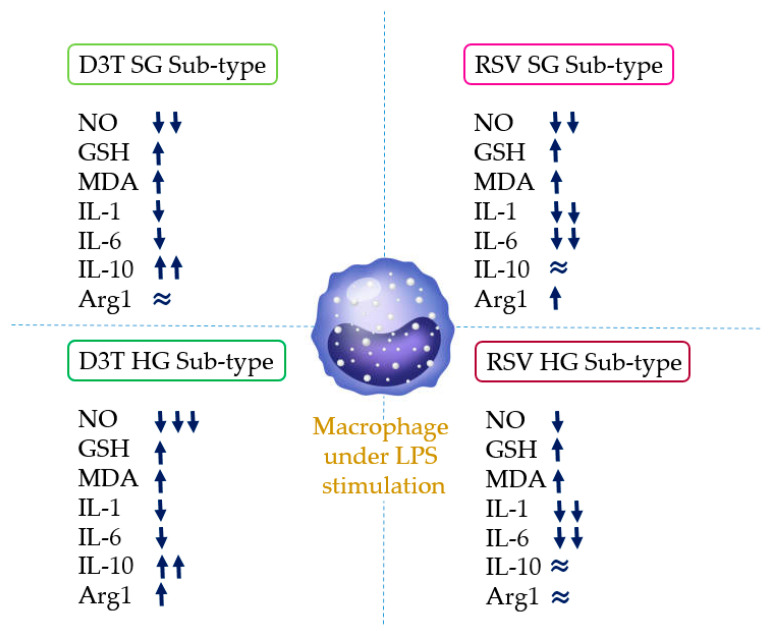
The potential effect of the bioactive compounds (D3T and RSV) over the RAW 264.7 macrophage phenotype under different concentrations of glucose (SG and HG) and LPS stimulation.

The present work demonstrates differences in the relative expression profiles of interleukins involved in both pro- and anti-inflammatory processes, as well as the status of endogenous antioxidant mechanisms (GSH) and the release of pro-inflammatory metabolites (NO and MDA) under SG and HG conditions, mainly under the stimulus of 60 ng/mL of LPS, by either D3T and RSV in RAW 264.7 macrophages. Classically, it is assumed that exposing macrophages to compounds classified as anti-inflammatory will result in M2 macrophages with similar effector characteristics, regardless of the compound tested. Our results suggest that this point of view would represent a bias in the potential outcomes, because other several factors, such as the concentration of glucose or elements potentially recognizable by the toll-type receptor 4 (TRL4), would affect the effective functionality of M2-polarized macrophages (Figure 9). Based on the evaluations shown in this study, we can conclude that different profiles of polarized M2 macrophages are achieved depending on a diverse number of stimuli. Overall, the results demonstrate that D3T-induced M2 macrophages have a greater capacity for the production of anti-inflammatory elements such as IL-10. However, RSV supplementation prompts macrophages with a greater capacity to inhibit the expression of pro-inflammatory elements such as interleukins 1 and 6, with low to null effects on anti-inflammatory elements. Despite the fact that RAW 264.7 macrophage cell line is a classic model for the evaluation of pro-inflammatory mechanisms, it would be highly relevant to evaluate these conditions in primary macrophage cultures, which could generate a better approximation of the effect of these bioactive compounds in the context of metabolic dysfunction. All these factors are important to take into consideration to generate more effective strategies for the treatment of inflammatory-related diseases using bioactive natural products, where macrophages play a fundamental role in the promotion of these processes.

## Data Availability

Not applicable.

## Supplementary Materials

The following supporting information can be downloaded at: https://www.mdpi.com/article/10.3390/molecules28165998/s1.

## References

[1] Pan J., Zhou L., Zhang C., Xu Q., Sun Y. (2022). Targeting protein phosphatases for the treatment of inflammation-related diseases: From signaling to therapy. Signal Transduct. Target. Ther..

[2] Mosser D.M., Edwards J.P. (2008). Exploring the full spectrum of macrophage activation. Nat. Rev. Immunol..

[3] Wang Y., Xu Y., Zhang P., Ruan W., Zhang L., Yuan S., Pang T., Jia A.-Q. (2018). Smiglaside A ameliorates LPS-induced acute lung injury by modulating macrophage polarization via AMPK-PPARγ pathway. Biochem. Pharmacol..

[4] Rathinam V.A., Chan F.K.-M. (2018). Inflammasome, inflammation, and tissue homeostasis. Trends Mol. Med..

[5] Meizlish M.L., Franklin R.A., Zhou X., Medzhitov R. (2021). Tissue homeostasis and inflammation. Annu. Rev. Immunol..

[6] Li C., Xu M.M., Wang K., Adler A.J., Vella A.T., Zhou B. (2018). Macrophage polarization and meta-inflammation. Transl. Res..

[7] Viola A., Munari F., Sánchez-Rodríguez R., Scolaro T., Castegna A. (2019). The metabolic signature of macrophage responses. Front. Immunol..

[8] Hotamisligil G.S. (2006). Inflammation and metabolic disorders. Nature.

[9] Napoli C., Paolisso G., Casamassimi A., Al-Omran M., Barbieri M., Sommese L., Infante T., Ignarro L.J. (2013). Effects of nitric oxide on cell proliferation: Novel insights. J. Am. Coll. Cardiol..

[10] Engström A., Erlandsson A., Delbro D., Wijkander J. (2014). Conditioned media from macrophages of M1, but not M2 phenotype, inhibit the proliferation of the colon cancer cell lines HT-29 and CACO-2. Int. J. Oncol..

[11] Kaur J. (2014). A comprehensive review on metabolic syndrome. Cardiol. Res. Pract..

[12] Yu B., Qin S.-y., Hu B.-l., Qin Q.-y., Jiang H.-x., Luo W. (2019). Resveratrol improves CCL4-induced liver fibrosis in mouse by upregulating endogenous IL-10 to reprogramme macrophages phenotype from M (LPS) to M (IL-4). Biomed. Pharmacother..

[13] Orecchioni M., Ghosheh Y., Pramod A.B., Ley K. (2020). Corrigendum: Macrophage polarization: Different gene signatures in M1 (LPS+) vs. classically and M2 (LPS–) vs. alternatively activated macrophages. Front. Immunol..

[14] Kang B., Kim C.Y., Hwang J., Suh H.J., Choi H.S. (2019). Brassinin, a phytoalexin in cruciferous vegetables, suppresses obesity-induced inflammatory responses through the Nrf2-HO-1 signaling pathway in an adipocyte-macrophage co-culture system. Phytother. Res..

[15] Tanase D.M., Gosav E.M., Anton M.I., Floria M., Seritean Isac P.N., Hurjui L.L., Tarniceriu C.C., Costea C.F., Ciocoiu M., Rezus C. (2022). Oxidative stress and NRF2/KEAP1/ARE pathway in diabetic kidney disease (DKD): New perspectives. Biomolecules.

[16] Kopacz A., Kloska D., Forman H.J., Jozkowicz A., Grochot-Przeczek A. (2020). Beyond repression of Nrf2: An update on Keap1. Free Radic. Biol. Med..

[17] Liu Z., Liao W., Yin X., Zheng X., Li Q., Zhang H., Zheng L., Feng X. (2020). Resveratrol-induced brown fat-like phenotype in 3T3-L1 adipocytes partly via mTOR pathway. Food Nutr. Res..

[18] Kuo P.-C., Yu I.-C., Scofield B.A., Brown D.A., Curfman E.T., Paraiso H.C., Chang F.-L., Yen J.-H. (2017). 3H-1, 2-Dithiole-3-thione as a novel therapeutic agent for the treatment of ischemic stroke through Nrf2 defense pathway. Brain Behav. Immun..

[19] Huang X., Li X., Xie M., Huang Z., Huang Y., Wu G., Peng Z., Sun Y., Ming Q., Liu Y. (2019). Resveratrol: Review on its discovery, pharmacokinetics and anti-leukemia effects. Chem. Biol. Interact..

[20] Poulsen M.M., Jørgensen J.O.L., Jessen N., Richelsen B., Pedersen S.B. (2013). Resveratrol in metabolic health: An overview of the current evidence and perspectives. Ann. N. Y. Acad. Sci..

[21] Pararasa C., Bailey C.J., Griffiths H.R. (2015). Ageing, adipose tissue, fatty acids and inflammation. Biogerontology.

[22] Pérez L.M., Pareja-Galeano H., Sanchis-Gomar F., Emanuele E., Lucia A., Gálvez B.G. (2016). ‘Adipaging’: Ageing and obesity share biological hallmarks related to a dysfunctional adipose tissue. J. Physiol..

[23] Nonnenmacher Y., Hiller K. (2018). Biochemistry of proinflammatory macrophage activation. Cell. Mol. Life Sci..

[24] Gerrick K.Y., Gerrick E.R., Gupta A., Wheelan S.J., Yegnasubramanian S., Jaffee E.M. (2018). Transcriptional profiling identifies novel regulators of macrophage polarization. PLoS ONE.

[25] Miao L., Shen X., Whiteman M., Xin H., Shen Y., Xin X., Moore P.K., Zhu Y.-Z. (2016). Hydrogen sulfide mitigates myocardial infarction via promotion of mitochondrial biogenesis-dependent M2 polarization of macrophages. Antioxid. Redox Signal..

[26] Zhao M., Li F., Jian Y., Wang X., Yang H., Wang J., Su J., Lu X., Xi M., Wen A. (2020). Salvianolic acid B regulates macrophage polarization in ischemic/reperfused hearts by inhibiting mTORC1-induced glycolysis. Eur. J. Pharmacol..

[27] Dissanayake W.C., Oh J.K., Sorrenson B., Shepherd P.R. (2021). Glucose regulates expression of pro-inflammatory genes, IL-1β and IL-12, through a mechanism involving hexosamine biosynthesis pathway-dependent regulation of α-E catenin. Biosci. Rep..

[28] Vasileva L.V., Savova M.S., Amirova K.M., Dinkova-Kostova A.T., Georgiev M.I. (2020). Obesity and NRF2-mediated cytoprotection: Where is the missing link?. Pharmacol. Res..

[29] Bigagli E., Cinci L., Paccosi S., Parenti A., D’Ambrosio M., Luceri C. (2017). Nutritionally relevant concentrations of resveratrol and hydroxytyrosol mitigate oxidative burst of human granulocytes and monocytes and the production of pro-inflammatory mediators in LPS-stimulated RAW 264.7 macrophages. Int. Immunopharmacol..

[30] Palacz-Wrobel M., Borkowska P., Paul-Samojedny M., Kowalczyk M., Fila-Danilow A., Suchanek-Raif R., Kowalski J. (2017). Effect of apigenin, kaempferol and resveratrol on the gene expression and protein secretion of tumor necrosis factor alpha (TNF-α) and interleukin-10 (IL-10) in RAW-264.7 macrophages. Biomed. Pharmacother..

[31] Zhu H., Bui A., Santo A., Li Y.R. (2022). 3 H-1, 2-dithiole-3-thione suppresses LPS-induced proinflammatory responses in macrophages: Potential involvement of antioxidant induction, NF-κB, and Nrf2. Mol. Cell. Biochem..

[32] Zhu H., Jia Z., Zhang L., Yamamoto M., Misra H.P., Trush M.A., Li Y. (2008). Antioxidants and phase 2 enzymes in macrophages: Regulation by Nrf2 signaling and protection against oxidative and electrophilic stress. Exp. Biol. Med..

[33] Klimontov V.V., Saik O.V., Korbut A.I. (2021). Glucose variability: How does it work?. Int. J. Mol. Sci..

[34] Saleh H.A., Ramdan E., Elmazar M.M., Azzazy H.M., Abdelnaser A. (2021). Comparing the protective effects of resveratrol, curcumin and sulforaphane against LPS/IFN-γ-mediated inflammation in doxorubicin-treated macrophages. Sci. Rep..

[35] Venter G., Oerlemans F.T., Wijers M., Willemse M., Fransen J.A., Wieringa B. (2014). Glucose controls morphodynamics of LPS-stimulated macrophages. PLoS ONE.

[36] Song M., Kellum J.A., Kaldas H., Fink M.P. (2004). Evidence that glutathione depletion is a mechanism responsible for the anti-inflammatory effects of ethyl pyruvate in cultured lipopolysaccharide-stimulated RAW 264.7 cells. J. Pharmacol. Exp. Ther..

[37] Cui F., Sequeira S.B., Huang Z., Shang G., Cui Q., Yang X. (2020). Bromosulfophthalein suppresses inflammatory effects in lipopolysaccharide-stimulated RAW264. 7 macrophages. Immunopharmacol. Immunotoxicol..

[38] Bonnefont-Rousselot D. (2016). Resveratrol and cardiovascular diseases. Nutrients.

[39] Zhou C., Zhang X., Ruan C.-C., Cheang W.S. (2021). Two methoxy derivatives of resveratrol, 3,3′,4,5′-tetramethoxy-trans-stilbene and 3,4′,5-trimethoxy-trans-stilbene, suppress lipopolysaccharide-induced inflammation through inactivation of MAPK and NF-κB pathways in RAW 264.7 cells. Chin. Med..

[40] Tsikas D. (2017). Assessment of lipid peroxidation by measuring malondialdehyde (MDA) and relatives in biological samples: Analytical and biological challenges. Anal. Biochem..

[41] Qureshi A.A., Guan X.Q., Reis J.C., Papasian C.J., Jabre S., Morrison D.C., Qureshi N. (2012). Inhibition of nitric oxide and inflammatory cytokines in LPS-stimulated murine macrophages by resveratrol, a potent proteasome inhibitor. Lipids Health Dis..

[42] Jayakumar T., Wu M.-P., Sheu J.-R., Hsia C.-W., Bhavan P.S., Manubolu M., Chung C.-L., Hsia C.-H. (2021). Involvement of antioxidant defenses and NF-κB/ERK signaling in anti-inflammatory effects of pterostilbene, a natural analogue of resveratrol. Appl. Sci..

[43] Chung S., Ranjan R., Lee Y.G., Park G.Y., Karpurapu M., Deng J., Xiao L., Kim J.Y., Unterman T.G., Christman J.W. (2015). Distinct role of FoxO1 in M-CSF-and GM-CSF-differentiated macrophages contributes LPS-mediated IL-10: Implication in hyperglycemia. J. Leukoc. Biol..

[44] Figueiredo R.D.A., Ortega A.C., Gonzalez Maldonado L.A., Castro R.D.d., Ávila-Campos M.J., Rossa Junior C., Aquino S.G.D. (2020). Perillyl alcohol has antibacterial effects and reduces ROS production in macrophages. J. Appl. Oral Sci..

[45] Vistica D.T., Skehan P., Scudiero D., Monks A., Pittman A., Boyd M.R. (1991). Tetrazolium-based assays for cellular viability: A critical examination of selected parameters affecting formazan production. Cancer Res..

[46] Strober W. (2015). Trypan blue exclusion test of cell viability. Curr. Protoc. Immunol..

[47] De Leon J.A.D., Borges C.R. (2020). Evaluation of oxidative stress in biological samples using the thiobarbituric acid reactive substances assay. JoVE (J. Vis. Exp.).

