# Supplementary material for: Macrophage Activity under Hyperglycemia: A Study of the Effect of Resveratrol and 3H-1,2-Dithiole-3-thione on Potential Polarization
Source: Molecules. 2023 Aug 10;28(16):5998. doi: 10.3390/molecules28165998 (PMC10458500; doi:10.3390/molecules28165998)
Supplement: Supplementary file 1 [file molecules-28-05998-s001.zip › molecules-2510138-supplementary.pdf]

molecules

MDPI

Supplementary Material

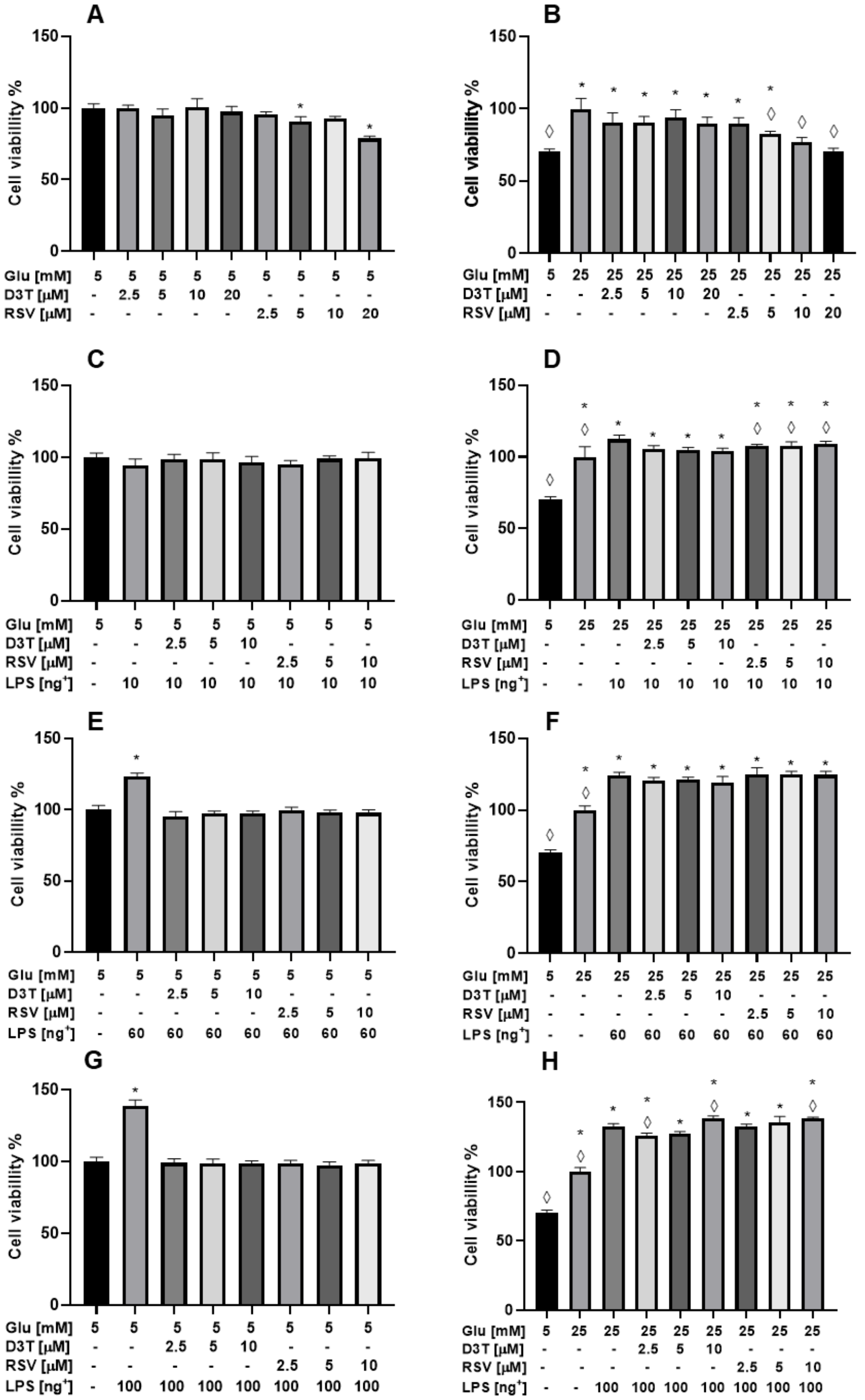

Figure S1. Effect of RSV and D3T supplementation on cell viability by the trypan blue assay of RAW 264.7 cell line under glucose and LPS stimulation. Macrophage viability under: A) SG without LPS, B) HG without LPS, C) SG with 10 ng/mL of LPS, D) HG with 10 ng/mL of LPS, E) SG with 60 ng/mL of LPS, F) HG with 60 ng/mL of LPS, G) SG with 100 ng/mL of LPS, H) HG with 100 ng/mL of LPS. * Indicates statistical difference respect to control SG (5 mM) in Figures A, C, E and G, and ◊ indicates statistical difference respect to HG without LPS in Figure B and HG stimulated with LPS in Figures D, F and H. + LPS concentration (ng/mL).

Table S1. Sequences of the primers used for the analysis of the relative expression of genes related to the macrophage polarization phenomenon.

Gene	Forward primer	Reverse primer	Reference
**Arg1**	CATTGGCTTGCGAGACGTAGAC	GCTGAAGGTCTCTTCCATCACC	NM_007482.3
**CYBB**	TCAGTGAGCTTTCCCTGTGTC	GCATTTGCCTTCGGTGATGT	NM_007807.5
**IL-1**	TGCCACCTTTTGACAGTGATG	AAGGTCCACGGGAAAGACAC	NM_008361.4
**IL-6**	CCACTTCACAAGTCGGAGGCTT	GCAAGTGCATCATCGTTGTTCATAC	NM_001314054.1
**IL-10**	GCCTTGCAGAAAAGAGAGCT	AAAGAAAGTCTTCACCTGGC	NM_010548.2
